# Impact of weight reduction on selected immune system response among Hepatitis C virus Saudi patients

**DOI:** 10.4314/ahs.v18i2.27

**Published:** 2018-06

**Authors:** Shehab M Abd El-Kader, Osama H Al-Jiffri

**Affiliations:** 1 Department of Physical Therapy, Faculty of Applied Medical Sciences, King AbdulazizUniversity, Jeddah, Saudi Arabia; 2 Department of Medical Laboratory Technology, Faculty of Applied Medical Sciences, King Abdulaziz University, Jeddah, Saudi Arabia

**Keywords:** Hepatitis C virus, obesity, immune system, weight reduction

## Abstract

**Background:**

Recently, about 2.35% of the world populations are estimated to be chronically infected with hepatitis C virus (HCV). Previous cohort studies indicated that obesity increases risk of hepatic steatosis and fibrosis in non-diabetic patients with chronic hepatitis C infection due to diminished response to anti-viral therapy and as a result obesity is considered as an important factor in the progression of chronic HCV. However, there is a strong association between BMI and the human immune system among HCV patients.

**Objective:**

This study aimed to examine effects of weight reduction program on selected immune parameters among HCV Saudi patients.

**Material and methods:**

One-hundred obese Saudi patients with chronic HCV infection participated in this study, their age ranged from 50–58 years and their body mass index (BMI) ranged from 30–35 kg/m^2^. All Subjects were included in two groups: The first group received weight reduction program in the form of treadmill aerobic exercises in addition to diet control whereas, the second group received no therapeutic intervention. Parameters of CD3, CD4 and CD8 were quantified; Leukocyte, differential counts and BMI were measured before and after 3 months, at the end of the study.

**Results:**

The mean values of BMI, white blood cells, total neutrophil count, monocytes, CD3, CD4 and CD8 were significantly decreased in the training group as a result of weight loss program; however the results of the control group were not significant. Also, there were significant differences between both groups at the end of the study.

**Conclusion:**

Weight loss modulates immune system parameters of patients with HCV.

## Introduction

Globally, an estimated 180 million people are chronically infected with HCV and 3 to 4 million are newly infected each year^1^,^2^. Hepatitis C virus (HCV) infection is one of the main causes of chronic liver disease worldwide^3^ and persistent infection occurs in 50 to 80% of those infected and may lead to the development of cirrhosis and subsequent hepatocellular carcinoma^1^.

The progression of HCV involves changes in the cellular immunity of those affected^4^,^5^. Some studies have indicated that the cellular immunity of HCV patients undergoes alterations, leading to poor immunological responses or dysfunctions^6^–^8^.

Obesity has also been associated with decreased immunocompetence as it alters innate and adaptive immunity and immunity deterioration is related to the grade of obesity^9^. Moreover, impaired immune responses have also been suggested to occur in obese humans. Studies indicated that the incidence and severity of certain infections are higher in obese individuals when compared to lean people^10^,^11^. Retrospective and prospective studies showed obesity to be an independent risk factor for infection after trauma^12^–^14^. In a prospective cohort study of critically ill trauma patients, obese patients had more than two fold increased risk of acquiring infection^12^. Also, Renehan et al. demonstrated an association of obesity with 25–40% of certain malignancies in both obese men and women^15^.

Many authors reported dysregulation and alteration in number of immune cells in obese subjects. Obese subjects showed either increased or decreased total lymphocytes in peripheral blood populations^16^–^19^ and had decreased CD8^+^ T cell population along with increased or decreased CD4^+^T cells^18^,^19^. Moreover, many previous studies as Moulin et al. who showed in their study that obesity is associated with the modulation of immune parameters^23^, elevated numbers of circulating immune cells as neutrophil, monocyte, leukocyte and total white blood cells (WBC)^16^,^24^, as well as elevated activation levels of certain WBC and suppressed immune cell function^17^. Also, several authors have reported a chronic inflammation status in individuals with higher BMI^25^–^27^ which was associated with elevated amounts of white blood cells, neutrophils, and monocytes in the blood of all participants with BMI higher than that of the control group^28^. Also, Kintscher et al. observed an increased number of CD3 and CD4 lymphocytes in the peripheral blood of obese women correlating with body mass index (BMI)^29^. Finally, Antuna-Puente et al. found that BMI is positively correlated with the number of macrophages in adipose tissue^30^.

As there are limited studies reporting the benefits of lifestyle modification on immune system response among hepatitis C virus Saudi patients. This study aimed to examine effects of a weight reduction program on selected immune parameters among HCV Saudi patients.

## Patients and methods

### Subjects

**Figure (1):**
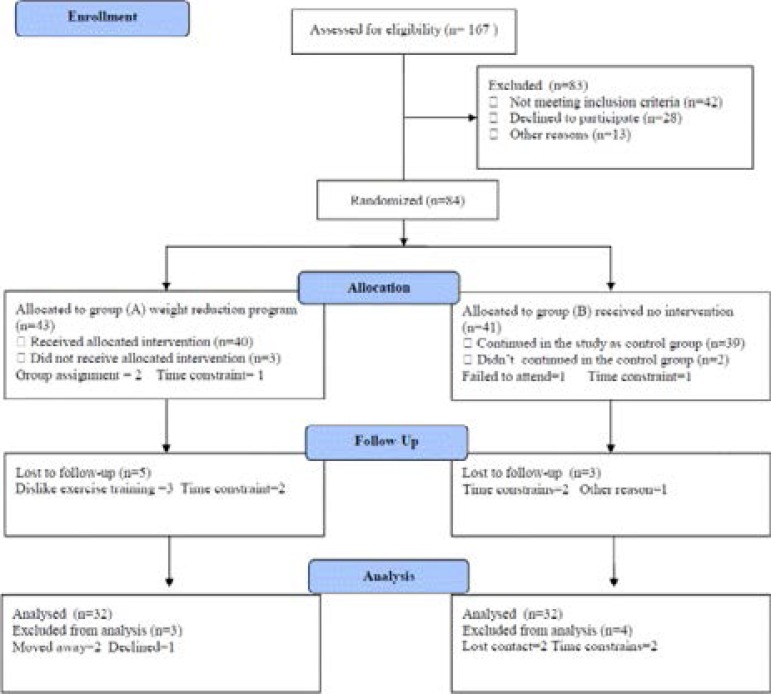
Subjects screening and recruitment CONSORT diagram.

One hundred non-hypertensive, non-cirrhotic Saudi patients with chronic HCV infection;their age ranged from 50 to 38 (41.63 ± 4.57) years, were selected on referral to Gastroenterology and Hepatology Department, King Abdulaziz University Teaching Hospital, Saudi Arabia. All participants were anti HCV positive by enzyme-linked immunosorbent assay (ELISA). Exclusion criteria included other potential causes of liver disease, such as alcoholism or autoimmune phenomena. Only patients diagnosed with chronic HCV mono-infection and had anti HCV antibodies by ELISA were selected to undergo Real-Time polymerase chain reaction (RT-PCR) and were treated with combined pegylatedinterferon--alfa (PEG-IFNα)-ribavirin therapy. All participants were free to withdraw from the study at any time. Patients were divided in to two equal groups: Group (A): received weight reduction program in the form of treadmill aerobic exercises in addition to diet control, Whereas group (B): received no therapeutic intervention. The CONSORT diagram outlining the details of the screening, run-in and randomization phases of the study and reasons for participant exclusion illustrated in figure (1). Informed consent was obtained from all participants. This study was approved by the Scientific Research Ethical Committee, Faculty of Applied Medical Sciences at King Abdulaziz University.

### Measurements

Clinical and laboratory analysis were performed by independent assessors who were blinded to group assignment and not involved in the routine treatment of the patients, however the following measurements were taken before the study and after 3 months at the end of the study:

**A. Real-Time polymerase chain reaction (RT-PCR):** Ten milliliter blood samples were collected from each participant at study entry. The blood samples were obtained using disposable needles and heparinized vacuum syringes and stored at −70°C until assayed. Serum samples of all participants were tested for Real-Time polymerase chain reaction (RT-PCR) to detect serum HCV RNA levels by polymerase chain reaction using the COBAS TaqMan HCV test, v2.0 (Roche Diagnostics, Indianapolis, NJ, USA).

**B. Analysis of peripheral blood cells:** The analysis of peripheral blood cells (e.g., total and differential count) was performed on a Beckman Coulter AcT 5diff hematology analyzer. The values are expressed in percentages and absolute numbers.

**C. Flow cytometry analysis:** The human leukocyte differentiation antigens CD3, CD4 and CD8 (Beckman Coulter, Marseille, France) Five microliters of appropriate monoclonal antibody was added to 50 µL of a wholeblood sample and incubated for 15 minutes at room temperature. Thereafter, the erythrocytes were lysed with 125 µL of a lysing solution, OptiLyse C, for 10 minutes. The reaction was stopped by the addition of 250 µL phosphate-buffered saline.The samples were analyzed by flow cytometry using Cytomics FC 500 and CXP software (Beckman Coulter).The leukocyte subsets were defined by forward- and side-scatter pattern. The negative control value was determined by a fluorescence background and antibody-nonspecific staining.

**D. Body mass index (BMI):** Weight and height scale (Metrotype -England) was used to measure weight and height to calculate the body mass index (BMI). Body mass index was calculated by dividing the weight in kilograms by the square of the height in meters (Kg/m^2^). According to the WHO classification, a BMI of <18.5 kg/m^2^ is under weight, 18.5–24.9 kg/m^2^ is normal 25–29.9 kg/m^2^ is overweight. A BMI of > 30 kg/m^2^ is classified as obese and this group is further divided into moderate obesity (30–34.9 kg/m^2^), severe obesity (35–39.9 kg/m^2^) and very severe obesity (≤40 kg /m2)^31^.

### Procedures

Following the previous evaluation , all patients were divided randomly into the following groups:

**1. The training group (Group A):** These were submitted to aerobic exercise training to complete a 12-week treadmill aerobic exercise (Enraf Nonium, Model display panel Standard, NR 1475.801, Holland) which was conducted according to recommendation of aerobic exercise application approved by the American College of Sports Medicine^32^. Training program included 5 minutes for warming-up in the form of range motion and stretching exercises, 30 minutes of aerobic exercise training with intensity equal 60–70% of the individual maximum heart rate followed by cooling down for 10 minutes ( on treadmill with low speed and without inclination). Participants had 3 sessions /week for 3 months with close supervision of physical therapist. Also, a dietician performed an interview-based food survey for all participants of group (A) for detection of feeding habits, abnormal dietary behavior and to prescribe the balanced low caloric diet^33^ that provided 1200 Kilocalories/day for 12 weeks. The same dietitian continuously monitored all participant caloric intakes through reviewing the detailed record of food intake every 2 weeks by the dietitian^34^,^35^.

**2. The control group (Group B):** received no intervention.

### Statistical analysis

The mean values of the investigated parameters obtained before and after three months in both groups were compared using paired “t” test. Independent “t” test was used for the comparison between the two groups (P<0.05).

## Results

The demographic and clinical characteristics of the subjects are shown in Table 1. There were no significant differences between both groups regarding age, height, albumin, fasting blood glucose, hemoglobin, total bilirubin, systolic blood pressure, diastolic blood pressure, body weight, body mass index (BMI), waist circumference, fat mass, alanine aminotransferase (ALT) and HCV viral.

**Table 1:** Comparison of clinical data between HCV patients in both groups

	Group (A)	Group (B)	Significance
**Age** (year)	50.21 ± 4.82	51.13 ± 3.27	P>0.05
**BMI** (kg/m^2^)	32.16 ± 2.25	31.71 ± 3.11	P>0.05
**Waist circumference** (cm)	94.33 ± 3.61	93.84 ± 4.53	P>0.05
**Fat mass** (kg)	25.10 ± 2.43	24.63 ± 2.31	P>0.05
**ALT** (U/L)	66.45 ± 7.28	65.37 ± 6.19	P>0.05
**Albumin** (gm/dl)	3.72 ± 0.92	3.46± 0.86	P>0.05
**FPG** (mg/dL)	113.91 ± 10.75	110.81 ± 8.42	P>0.05
**Hb** (gm/dl)	11.93 ± 1.64	12.16± 1.76	P>0.05
**Total Bilirubin** (mg/dl)	1.41 ± 0.82	1.39 ± 0.75	P>0.05
**SBP** (mm Hg)	125.42 ± 12.41	122.63 ± 10.33	P>0.05
**DBP** (mm Hg)	84.70 ± 6.57	83.95 ± 7.48	P>0.05
**HCV viral load** (IU/mL)	7.62 ± 3.81 × 10^6^	7.31 ± 3.94 × 10^6^	P>0.05

BMI: Body Mass Index; Hb: Hemoglobin; FPG: Fasting Blood Glucose; ALT: Alanine aminotransferase; SBP: Systolic blood pressure;DBP: Diastolic blood pressure.

**Figure (2):**
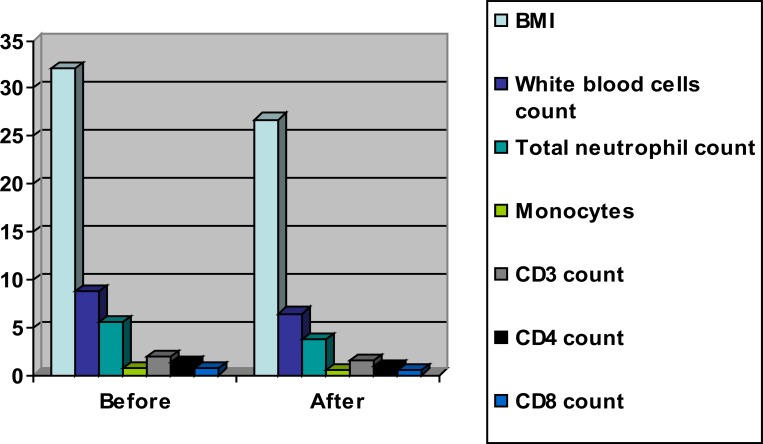
Mean value of body mass index, white blood cells, total neutrophil, monocytes, CD3, CD4 and CD8 count of group (A) before and at the end of the study.

**Figure (3):**
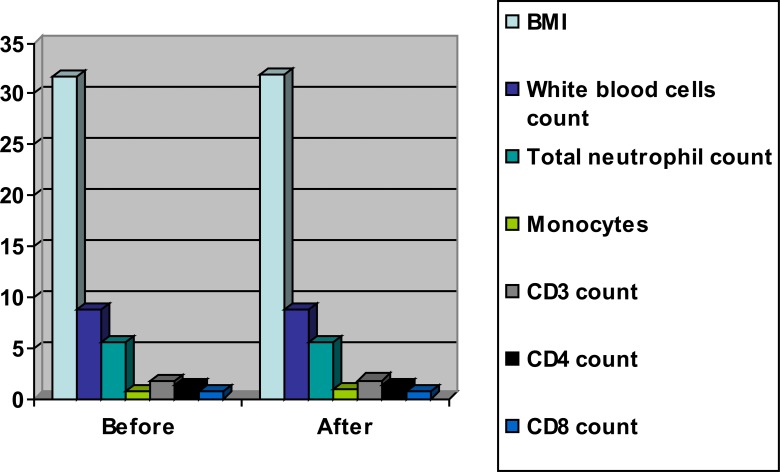
Mean value of body mass index, white blood cells, total neutrophil, monocytes, CD3, CD4 and CD8 count of group (B) before and at the end of the study.

**Figure (4):**
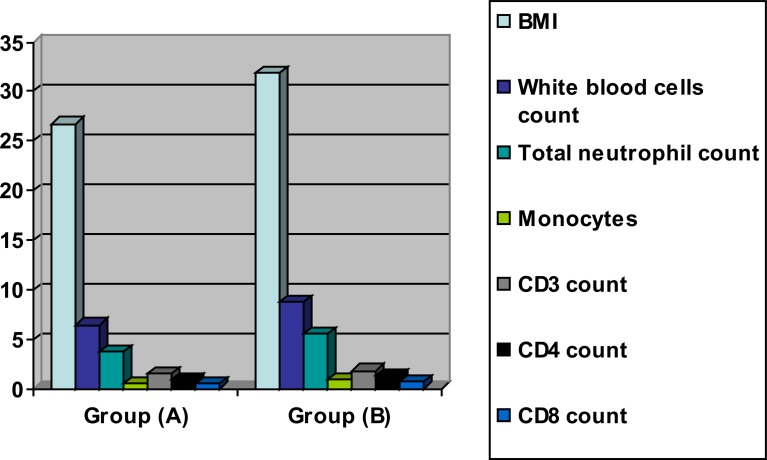
Mean value of body mass index, white blood cells, total neutrophil, monocytes, CD3, CD4 and CD8 count of group (A) and group (B) at the end of the study.

The mean values of BMI, white blood cells, total neutrophil count, monocytes, CD3, CD4 and CD8 were significantly decreased in the training group as a result of weight loss program (Table 2 and figure 2), however the results of the control group were not significant (table 3 and figure 3). Also, there were significant differences between both groups at the end of the study (table 4 and figure 4).

**Table 2:** Mean value and significance of body mass index, white blood cells, total neutrophil, monocytes, CD3, CD4 and CD8 count of group (A) before and at the end of the study

	Mean +SD		T-value	Significance
	Pre	Post		
**BMI** (kg/m^2^)	32.16 ± 2.25*	26.75 ± 2.12	8.24	P<0.05
**white blood cells** **count** (10^9^/µL)	8.73 ± 2.54*	6.42 ±2.43	7.82	P<0.05
**total neutrophil count** (10^9^/µL)	5.61 ±1.81*	3.74 ±1.59	7.23	P<0.05
**Monocytes** (10^9^/µL)	0.71 ± 0.25*	0.48 ±0.16	5.41	P<0.05
**CD3 count** (10^9^/L)	1.85 ± 0.92*	1.46 ±0.71	6.52	P<0.05
**CD4 count** (10^9^/L)	1.34 ± 0.73*	0.92 ± 0.64	6.25	P<0.05
**CD8 count** (10^9^/L)	0.72 ± 0.23*	0.45 ± 0.12	5.17	P<0.05

BMI= Body Mass Index

* indicates a significant difference between the two groups, P < 0.05

**Table 3:** Mean value and significance of body mass index, white blood cells, total neutrophil, monocytes, CD3, CD4 and CD8 count of group (B) before and at the end of the study

	Mean +SD		T-value	Significance
	Pre	Post		
**BMI** (kg/m^2^)	31.71 ± 3.11	31.85 ± 3.06	0.853	P>0.05
**white blood cells** **count** (10^9^/µL)	8.69 ± 2.35	8.81 ± 2.42	0.742	P>0.05
**total neutrophil** **count** (10^9^/µL)	5.47 ±1.66	5.53 ±1.68	0.615	P>0.05
**Monocytes** (10^9^/µL)	0.74 ± 0.27	0.81 ± 0.29	0.378	P>0.05
**CD3 count** (10^9^/L)	1.62 ± 0.85	1.79 ± 0.87	0.432	P>0.05
**CD4 count** (10^9^/L)	1.27 ± 0.64	1.32 ± 0.66	0.419	P>0.05
**CD8 count** (10^9^/L)	0.68 ± 0.21	0.71 ± 0.22	0.326	P>0.05

BMI= Body Mass Index

* indicates a significant difference between the two groups, P < 0.05

**Table 4:** Mean value and significance of body mass index, white blood cells, total neutrophil, monocytes, CD3, CD4 and CD8 count of group (A) and group (B) at the end of the study

	Mean +SD		T-value	Significance
	Group (A)	Group (B)		
**BMI** (kg/m^2^)	26.75 ± 2.12*	31.85 ± 3.06	7.21	P<0.05
**white blood cells** **count** (10^9^/µL)	6.42 ±2.43*	8.81 ± 2.42	6.39	P<0.05
**total neutrophil count** (10^9^/µL)	3.74 ±1.59*	5.53 ±1.68	6.14	P<0.05
**Monocytes** (10^9^/µL)	0.48 ±0.16*	0.81 ± 0.29	4.31	P<0.05
**CD3 count** (10^9^/L)	1.46 ±0.71*	1.79 ± 0.87	5.45	P<0.05
**CD4 count** (10^9^/L)	0.92 ± 0.64*	1.32 ± 0.66	5.28	P<0.05
**CD8 count** (10^9^/L)	0.45 ± 0.12*	0.71 ± 0.22	4.36	P<0.05

BMI= Body Mass Index

* indicates a significant difference between the two groups, P < 0.05.

## Discussion

The best immune correlate of HCV control is a strong and broad CD4^+^ T-cell response to HCV antigens, which is often associated with an equally robust and broad HCV specific CD8^+^ T-cell immune response^36^–^38^. It is believed that CD4^+^ T cells are necessary to ensure fully functional CD8^+^ T-cell responses, which then can clear the virus^39^. However, obesity is associated with the modulation of immune parameters^40^,^41^. To our knowledge, this is the first study of immune function measures in relation to previous intentional weight loss in HCV patients. Our results indicate that there may be long-term effects of intentional weight loss on immune function. These results are in line with several previous studies.

Shade and colleagues conducted a study on one hundred fourteen healthy, overweight, sedentary, post-menopausal women, who were recruited for an exercise intervention study and were currently weight stable who lost ≥ 10 pounds had lower measured natural killer cell (NK) cytotoxicity than those who did not (24.7%±12.1% vs 31.1%±14.7%, respectively). The frequency of weight loss episodes was also associated with differences in the number and proportion of NK cells. This study provides evidence that frequent intentional weight loss may have long-term effects on immune function^42^. However, Wasinski and colleagues conducted a study on mice who were submitted to chronic swimming training or a 30% caloric restriction after consuming a high-fat diet. The mice were subjected to swimming sessions 5 times per week for 6 weeks.Data demonstrated that exercise and caloric restriction modulate resident immune cells in adipose tissues. We observed a reduction in CD4^+^ and CD8^+^ T lymphocytes in AT^43^. Also, Carpenter and colleagues evaluated the forced and voluntary exercise as weight-loss treatments in diet-induced obese (DIO) mice and assessed the effects of weight loss on monocyte concentration and cell-surface expression of Toll-like receptor. Results confirmed that short-term exercise and low-fat diet consumption over the 8 weeks caused significant weight loss and altered immune profiles^44^. While, Viardot and colleagues looked at 13 obese people with Type 2 diabetes or pre-diabetes who were limited to a diet of between 1000 and 1600 calories a day for 24 weeks. Gastric banding was performed at 12 weeks to help restrict food intake further. Their results showed an 80% reduction of pro-inflammatory T-helper cells, as well as reduced activation of other circulating immune cells (T cells, monocytes and neutrophils) and decreased activation of macrophages in fat^45^. Wing and colleagues examined subjects after weight loss, induced by 14-days fast, showed improvement in serum immunoglobulin levels, bactericidal capacity of blood monocytes and NK cell cytotoxic activity^46^. Moreover, Tanaka and colleagues reported that caloric restriction improves parameters of immunity such as T cell counts and NK cell activity and the ability of mononuclear cells to produce pro-inflammatory cytokines^47^.

The possible mechanism of immune system modulation by weight reduction could be explained by reduction of adipose tissues which is not only a storage organ, but produces close to 100 cytokines. These secreted adipokines are directly correlated to the increased adipose tissue mass and play an intricate role in various aspects of the innate and adaptive immune response and participate in a wide variety of physiological or physiopathological processes including food intake, insulin sensitivity and inflammation^48^. Also, weight reduction reduces serum level of leptin. Leptin has pleiotropic effects on immune cell activity as evidenced from the presence of leptin receptors on all immune cells of both the arms of innate and adaptive immunity^49^. Leptin promotes macrophage phagocytosis by activating phospholipase. Leptin increases secretion of pro-inflammatory cytokines by macrophages. Leptin stimulates monocyte proliferation andupregulates the expression of activation markers including CD38, CD69, CD25 and CD71^50^. Leptin is involved in the natural killer cell development, differentiation, proliferation, activation and cytotoxicity^51^.

The current study has important strengths and limitations. The major strength is the supervised nature of the study. Supervising food intake and physical activity removes the need to question compliance or to rely on food and activity questionnaires. Further, all exercise sessions were supervised and adherence to the diet and activities was essentially 100%. Moreover, the study was randomized; hence, we can extrapolate adherence to the general population. In the other hand, the major limitation is the small sample size in both groups which may limit the possibility of generalization of the findings in the present study. Finally, within the limit of this study, weight reduction is recommended for modulation of immune system parameters of patients with HCV. Further researches are needed to explore the impact of weight reduction on quality of life and other biochemical parameters among patients with HCV.

## Conclusion

The current study provides evidence that weight loss modulates immune system parameters of patients with HCV.
